# Partial Two-Stage Exchange for Infected Total Hip Arthroplasty: A Treatment to Take into Account

**DOI:** 10.3390/jpm13010137

**Published:** 2023-01-10

**Authors:** Miguel Moreno-Romero, Alejandro Ordas-Bayon, Alejandro Gomez-Rice, Miguel A. Ortega, Basilio J. De La Torre Escuredo

**Affiliations:** 1 Department of Orthopaedic Surgery, University Hospital Ramón y Cajal, 28034 Madrid, Spain; 2 Department of Medicine and Medical Specialities, Faculty of Medicine and Health Sciences, University of Alcalá, 28801 Alcalá de Henares, Spain; 3 Ramón y Cajal Institute of Sanitary Research (IRYCIS), 28034 Madrid, Spain; 4 Department of Surgery, Medical and Social Sciences, Faculty of Medicine and Health Sciences, University of Alcalá, 28801 Alcalá de Henares, Spain

**Keywords:** infection, hip, arthroplasty, partial, exchange

## Abstract

Introduction: Two-stage revision is the gold standard for chronic periprosthetic joint infection (PJI). The removal of well-fixed implants, especially the femoral component, can be extremely difficult and additional osteotomies may be needed, which is time-consuming and results in bone stock loss. When the femoral stem is osseointegrated, there is no clear indication for the use of partial two-stage revision. The primary objective was to assess infection eradication after surgery. Methods: Retrospective study of specific case series. A total of eight patients with a chronic uncemented PJI, in the setting of complex revision surgeries, were treated with partial two-stage revision, which included selective retention of the well-fixed femoral component and complete acetabular removal. Stem retention was carried out regardless of the bacteria or associated comorbidities. Results: All patients were re-revision cases with at least two previous surgeries (range, 2–4). Complex revisions were performed in five cases (non-articulated spacer) and simple revisions in three cases (articulated spacer). The minimum follow-up time was 24 months (range, 24–132 months). The infection eradication rate at final follow-up was 100%. Conclusion: Partial two-stage reconstruction is a promising technique for the treatment of chronic PJI in patients with a well-fixed stem and complex re-revision acetabular procedures. Further prospective studies and prolonged follow-ups are required to confirm our results.

## 1. Introduction

Periprosthetic joint infection (PJI) of the hip joint is one of the most severe complications in total hip replacement (THR) surgery. Its incidence oscillates between 0.3 and 2.2% in primary THR [1], and between 3 and 4% in revision THR [2], and the final cost of the treatment represents a real health emergency from a purely economic point of view [3]

Chronic PJI is a surgical challenge for the orthopaedic surgeon, two-stage revision being the gold standard treatment [4]. Reported success rates, defined as infection eradication, vary from 90 to 100% [5]. The main disadvantage of this procedure is that it requires two surgeries. Alternatively, one-stage revision is becoming more popular due to comparable outcomes with less surgical aggression [6].

Surgical decision-making becomes more challenging when treating chronic hip PJI with osseointegrated implants. This becomes an even more complex issue when revision implants have previously been used.

The removal of well-fixed implants implies additional morbidity, as it leads to significant bone loss and makes revision surgery more difficult [7,8]. Lately, some case series have reported partial revision surgery, meaning retention of well-fixed implants and removal of loose components.

The aim of our study is to the report the long-term outcomes of a specific case series of patients with complex infected revision surgeries treated with partial revision surgery, with the ultimate purpose of infection eradication. Our results are also compared to similar studies in order to establish a protocol that helps with decision-making in these complex cases.

## 2. Patients and Methods

We report a retrospective case series study that identified a consecutive series of patients who underwent partial revision surgery for complex chronic infected revision. Informed consent was obtained from all individual participants included in the study.

Patients were included if they met all three of the following criteria:

1. Uncemented THR and late chronic infection according to McPherson et al.’s [9] classification and the 2011 Musculoskeletal Infection Society (MSIS) criteria [10].
2. Well-fixed femoral component.
3. Two-year minimum follow-up.

A total of 45 patients with late chronic PJI following THR were identified in our database from September 2011 to May 2022. Out of 45 cases, 35 had a femoral stem septic loosening and were treated with total revision surgery in two stages; 10 patients had a well-fixed and osseous integrated femoral stem and underwent partial two-stage revision. Just 2 of these patients did not meet the two-year follow-up criteria. Finally, 8 patients were included in the study group. Demographic characteristics are summarized in Table 1.

For the purpose of this study, a “well-fixed femoral stem” was defined according to radiographic and intraoperative criteria. From a radiographic point of view, stems were considered loose in the presence of one of the following: subsidence greater than 2 mm, complete radiolucent line along the stem surface greater than 2 mm and endosteal scalloping or migration of the prosthesis [11]. Intraoperatively, femoral stems were considered osseointegrated if the implant could not be removed without the aid of osteotomy [2].

The diagnosis of deep periprosthetic joint infection of the hip was based on the criteria of the Musculoskeletal Infection Society [10], Table 2.

According to the above-mentioned criteria, 8 patients were treated with retention of their cementless stem: 6 patients had a diaphyseal support stem and 2 patients had primary stems.

### Patient Management and Surgical Technique

A single surgeon (BTE) operated on all patients. A posterolateral approach to the hip joint was used in all patients. A thorough debridement was performed, and samples were collected. After that, removal of the acetabular component was conducted. In some cases, purpose-built components such as the Explant Acetabular Cup Removal System (Zimmer, Biomet, Warsaw, IN) were used.

The subsequent step was to assess the fixation of the femoral component. When a modular revision stem had been used, the technique included disassembly of the two components, removing the metaphyseal component with the aid of specific removal devices. In order to assess whether the femoral component was well fixed, thin flexible osteotomes were used around the stem in the metaphyseal area for primary stems and in the diaphyseal area for modular revision stems.

**Figure 1:**
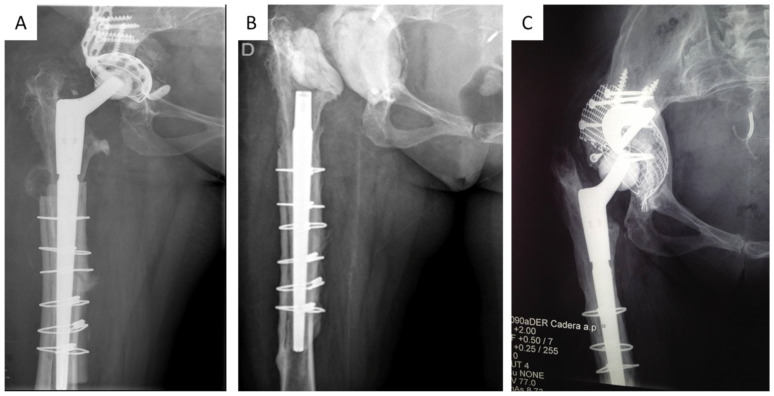
(**A**), Preoperative radiograph before the first stage in a patient with septic loosening of acetabular component; (**B**), radiograph with a non-articulated spacer; (**C**), radiograph at 9-year follow-up after the second stage with bone impaction grafting and trabecular metal augments (BIG-TMT).

After that, a pulsatile lavage of the exposed parts of the stem was carried out using 12 L of a combined solution of saline and povidone-iodine (Betadine). Finally, an antibiotic-loaded cement spacer was placed. The use of an articulating or a non-articulating spacer was chosen based on the extent of the acetabular defect and the type of femoral stem. For severe acetabular defects and in patients with revision modular stems—following the extraction of the metaphyseal component—non-articulated spacers were used (Figure 1B).

The PMMA used was medium-viscosity cement (PALACOS, Heraeus Medical, Yardley, PA) loaded with 2 g of Vancomycin and 1 g of Gentamycin per 40 g of cement. Additionally, and in agreement with the Infectious Diseases Department, IV antibiotic therapy was administered to every patient for two weeks, followed by six weeks of oral antibiotics.

Second-stage reconstruction was performed once all inflammatory markers were normalized or showed a downward trend two months after cessation of antibiotic treatment. During the second stage, a thorough debridement and washout was repeated. Sample collection with intraoperative pathology was performed. Once both the biopsy and Gram-stain were negative, we proceeded with the reconstruction, which was performed with varied techniques. In minor defects, an uncemented revision acetabular component was used (TMT Zimmer-Biomet). In severe defects, we used either trabecular metal augments with revision shell, cup–cage reconstruction, or a combination of trabecular metal supplements and morselized bone graft (Figure 1A–C).

After implantation of the components, IV teicoplanin and ertapenem were initiated for 5–7 days until microbiologic cultures were deemed negative.

Hip status was clinically assessed using the Western Ontario and McMaster Universities (WOMAC) questionnaire [12] according to preoperative questionnaires and the last follow-up. Its usefulness comes from its ability to assess clinical changes patients have perceived in their state of health.

The cases were also radiologically assessed by using pelvis AP and lateral hip views. Revision shell fixation and the metal trabecular augment reconstruction were assessed by using Moore’s method [13], while Gill’s method [14] was used for radiological evaluation of the cage.

Follow-up visits were carried out in conjunction with the Infectious Diseases Department. Laboratory C-reactive protein (CPR) tests were performed regularly to check recurrence of infection.

A successful treatment was defined as the absence of clinical symptoms and signs of infection. Treatment failure was defined as infection recurrence by the same bacteria isolated before the first stage surgery.

The authors affirm that the human research participants provided informed consent for publication of their clinical results and also the clinical images in Figure 1A–C.

## 3. Results

All eight patients were followed up for a minimum of 24 months (range, 24–132 months). In five patients, the acetabular cup was loose and easily removed. In the other three patients, since the acetabular component was fixed, we used the Explant Acetabular Cup Removal System (Zimmer, Warsaw, IN, USA).

A modular revision stem system had been used in six out of eight patients. For these six patients, removal of the metaphyseal component was planned. This was successfully achieved in five patients. In one patient (case number 3), the diaphyseal component could not be disengaged from the diaphyseal component.

Between the first and second stage, three patients had an articulated spacer over the cone and the metallic head of the femoral stem left in situ. In the remaining five patients, a non-articulated spacer was used.

The mean time from the first to the second stage was 23,5 weeks (range 10–64 weeks).

For the acetabular reconstruction, three cases with minor defects underwent TMT with revision shell (cases 3, 5 and 8). Case 1 had a combination of trabecular metal supplements and morselised bone graft, as reported previously by the senior author [15]. In three cases (cases 2, 4 and 6), all of them with pelvic discontinuity, the reconstruction was performed with fixation of the superior to the inferior hemipelvis by using pelvic reconstruction plating in combination with trabecular metal supplements and cup–cage construction. In case 7, reconstruction was performed by using revision shell plus trabecular metal augments (Table 3).

WOMAC pain, function and stiffness improved for every patient at the last follow-up (Table 4). The results were clinically relevant.

No component loosening was observed radiographically, confirming osseointegration [16]. Up to now, all eight patients have shown normalization of the inflammatory markers, taking CRP < 5 mg/dl as a normal value and ESR <20 mm/h (Table 5). Postoperative complications occurred in two patients (cases 6 and 7). Case 6, a 48-year-old female who sustained three dislocations, underwent the acetabular reconstruction with a cup–cage construction. The first episode occurred immediately after surgery, the second ten months after surgery, and the third four years later. All of them were treated with closed reduction. After discussing the different treatment alternatives with the patient, and assessing the complex reconstruction she had, we recommended conservative treatment with a hip abduction orthosis. She suffers from certain restriction due to instability fear. No other surgical complications were observed after the second stage. Case 7 was reconstructed with TMT revision shell and TM augments, and she suffered multiple dislocations. A revision surgery was carried out to modify the orientation of the metaphyseal component.

## 4. Discussion

In the presence of a chronic PJI with loose implants, total revision surgery is the gold standard treatment. The problem arises when any of the components are completely osseointegrated. The extraction of these components may be extremely difficult. This is particularly true in revision femoral stems, where the stem is osseointegrated in its full extension. Its removal frequently requires many techniques [17], the trochanteric osteotomy being the most extended, which has many potential drawbacks [8]. In this scenario, we decided to use the partial two-stage revision, leaving intact the femoral component. Many authors had reported their results with this technique [2,18,19,20,21,22,23,24,25,26,27], in some cases removing the acetabular components, in others the femoral component. In our series, the femoral stem was well fixed, and the acetabular component was removed in every case. Acetabular component extraction is eased nowadays by commercially available devices such as Explant (Zimmer Biomed, Warsaw, EEUU), which minimizes bone stock loss. The main findings of our work, in those selected patients in which we perform this technique, is that the infection eradication rate is similar than the traditional total exchange techniques.

We excluded from our study chronic PJI with well-fixed cemented stems from hip hemiarthroplasties, as, in this aspect, the literature shows contradictory outcomes, from excellent [28] to poor [29].

Prior to this study, we found 13 references concerning partial replacement. seven of them reported an isolated exchange of the acetabular component [18,19,20,21,25,26,27], while in the remaining six, either the femoral or acetabular component were exchanged. In our opinion, there is no point in retaining an acetabular component due to its relative ease of extraction.

Osseointegration may act as a barrier to bacterial colonisation and biofilm formation [30,31]. A particularly controversial issue is the exposed surfaces of the femoral stem, such as the neck, where biofilm could form [32]. However, several strategies are currently used to treat PJI after PTA, such as debridement, antibiotic pearls, hydrogels, nanoparticles with bactericidal effects and antimicrobial peptides [33]. Additionally, antiseptic solutions such as acetic acid or povidone-iodine have been shown to inhibit bacterial growth and are all effective and promising strategies to prevent explantation of the implanted component. This is the rationale for its use in our practice.

Once the second stage was complete, we prescribed systematic IV antibiotic therapy for five days until the cultures results were available, as reported by Shi et al. [34]. Unfortunately, there is no standard practice in partial replacement. Some authors do not mention this aspect [18,19,25], others use a 24-hour treatment after reimplantation [2] and some others even choose prolonged antibiotic administration, up to 2 weeks IV and 6 months orally [20].

Another crucial aspect of this technique is the lack of consensus concerning patient selection criteria. Some authors advocate for the exclusion of patients with important comorbidities [2], those presenting a sinus or when the causal bacteria have not been identified [25]. We decided to apply only criteria based on the osseointegration of the femoral stem. One of our patients had a chronic renal failure and active HIV and Hep-B active infections. Contrary to some opinions [23], in our series, the criteria for performing a partial two-stage revision were based on the osseointegration of the femoral stem, both for primary and revision stems.

Although in previous studies osseointegration of the stem was evaluated solely by the use of imaging techniques [2,34], we strongly advocate for intraoperative testing. We must bear in mind that, unfortunately, there is no standardized imaging protocol for definitely diagnosing femoral stem loosening [35].

As opposed to previous studies [2,20,34], we performed this technique with a known resistant bacteria infection (e.g., MRSA), which is a common cause of failure of revision replacement surgery [36]. We believe that the main cause for failure is not exclusively dependent on the type of replacement performed (total or partial), but intrinsic and extrinsic patient factors. One of them may be adequate antibiotic therapy, both local and systemic.

Previous studies reported infection eradication success rates that vary from 78% [23] to 100% [25,34] (Table 6). Those who did not reach a 100% success rate did not highlight any reason for it. Nevertheless, when analyzing the characteristics of those patients, common factors such as severe comorbidities [26] or resistant bacteria infection were found [2,37].

The rationale to develop this technique was to prevent further surgical aggression to the patient when the stem is osseointegrated. In the same manner, the main advantage of one-stage revision over two-stage revision is the possibility of resolving the problem with just one surgery [6]. Both El-Husseiny et al. in 2016 [38] and Ji et al. in 2017 [37] proposed partial revision surgery in one stage, with reported success rates over 80% with a minimum five-year follow-up. Therefore, this treatment seems to lead to a significant decrease in surgical aggression with comparable success on infection eradication. Consequently, we believe this option could be prioritised in a near future in patients presenting mild to moderate acetabular defects. In severe defects, we think that the two-stage partial revision is best indicated due to the role of the cement spacer as a carrier for antibiotics.

The main limitation of our study is the small number of cases and the short follow-up time compared to the standard revision techniques, which have been used for decades. Although the number of cases is a limitation, we believe that the case series is very specific, since it includes complex revision surgeries, many of them with large acetabular defects and a long follow-up. Nevertheless, considering previously published papers, adding up to a total of 268 cases, the reported success rate of this technique is 80%, which makes this technique comparable to one- or two-stage total revision replacements [39].

## 5. Conclusions

Partial hip revision surgery seems to be a safe procedure that significantly lessens surgical trauma. Despite the lack of consensus in patient selection criteria, the two key aspects for its indication are the osseointegration of the femoral stem and aggressive surgical debridement. If those conditions are present, two-stage partial hip revision surgery is a valid alternative for chronic hip PJI, particularly in the setting of severe acetabular defects, where the antibiotic delivery role of the spacer is critical for infection control.

## Figures and Tables

**Table 1:** Demographic characteristics. F: female; M: Male; HIV: human immunodeficiency virus; HCV: hepatitis C virus.

	Gender/Age at Surgery	Primary or Revision/Stem	Morbidities	PJI [9]
Case 1	F/44 years	Revision/Restoration Modular (Stryker)		III-A-2
Case 2	F/74	Revision/Arcos Modular (Zimmer-Biomet)	Hypertension	III-A-1
Case 3	M/64	Revision/Modular Revision (Lima)	Hypertension Diabetes Obesity	III-C-2
Case 4	F/57	Primary/Poropalcar (I.Q.L. Spain)	HIV IV drug user HCV	III-C-3
Case 5	F/72	Primary/Furlong HA (MBA)		III-A-2
Case 6	F/48	Revision/Arcos Modular (Zimmer-Biomet)	Smoker Obesity	III-B-2
Case 7	F/57	Revision/Arcos Modular (Zimmer-Biomet)	Smoker Obesity Kidney disease	III-C-2
Case 8	M/70	Revision/Arcos Modular (Zimmer-Biomet)	Hypertension Obesity	III-B-2

**Table 2:** Diagnosis of periprosthetic joint infection. CRP: C-reactive protein; ESR: erythrocyte sedimentation rate; WBC: white blood cell count; AP: anatomic pathology; MR: methicillin-resistant; MS methicillin-susceptible.

	Fistula	CRP ESR	WBC	Arthrocentesis	AP	Preoperative Culture	Intraoperative Culture
Case 1	−	+/+	+	+	+	*S. epidermidis* (MR)	*S. epidermidis* (MR)
Case 2	−	+/+	+	+	+	*Streptococcus mutans*	*Streptococcus. mutans*
Case 3	+	+/+	+	−	+	−	*S. epidermidis* (MR)
Case 4	−	+/+	+	+	+	*S. aureus* (MS)	*S. aureus* (MS)
Case 5	+	+/+	+	+	+	*P. aeruginosa*	*P. aeruginosa*
Case 6	+	+/+	+	+	+	*Morganella* *morgagnii*	*Morganella morgagnii*
Case 7	−	+/+	+	+	+	*S. epidermidis* (MR)	*S. epidermidis*
Case 8	+	+/+	+	+	+	*S. agalactiae*	*S. agalactiae*

**Table 3:** Intraoperative and postoperative features. NA: non-articulated; A: articulated; B.I.G: bone impaction grafting.

Case	Preop X-ray Acetabular Loosening	Acetabular Defects [14]	Spacer	Acetabular Reconstruction	Antibiotics Therapy (Weeks)	Time to 2nd Stage (Weeks)	Postop Cultures	Follow-Up (Months)
1	+	IV	NA	TMT + B.I.G.	8	20	−	132
2	+	V	NA	PLATE + CUP–CAGE	8	10	−	84
3	−	II	A	REVISION SHELL	8	16	−	96
4	+	V	A	PLATE + CUP–CAGE	8	64	−	84
5	−	II	A	REVISION SHELL	8	12	−	48
6	+	IV	NA	PLATE + CUP–CAGE	8	44	−	84
7	−	IV	NA	REVISION SHELL + TMT	8	12	−	36
8	−	II	NA	REVISION SHELL	8	10	−	24

**Table 4:** WOMAC scores.

	Preoperative Score Median	Postoperative Score Median	Difference
Pain	9	2	7
Stiffness	4	2	2
Function	29	18.5	10.5

**Table 5:** Inflammatory markers CRP/ESR before first stage (1st), second stage (2nd) and follow-up (F.U.).

	1st	2nd	F.U.	1st	2nd	F.U.
Case 1	103.2	46.7	4.3	34.3	25.3	19.4
Case 2	76.7	34.3	4.8	56.2	28.2	18.7
Case 3	282.4	67.8	3.1	102.2	22.4	15.4
Case 4	62.9	28.2	2.9	45.3	30.1	8.3
Case 5	93.9	54.2	5.3	48.9	22.3	15.6
Case 6	89.2	47.3	4.2	39.7	24.5	20.1
Case 7	101.3	31.4	3.1	42.4	27.4	18.4
Case 8	253.3	89.4	2.7	45.6	26.4	15.7

**Table 6:** Infection eradication rate.

Author	N	Follow-Up (Months)	Success Rate
Faroug et al.	2	39 (36–42)	100%
Anagnostakos et al.	13	55 (12–83)	91.60%
Lee et al.	19	48 (24–96)	88.20%
Ekpo et al.	19	48 (24–132)	89.50%
Lombardi et al.	26	19 (4–36)	85.70%
Fukui et al.	5	50 (42–60)	100%
Baochao et al.	31	60 (24–180)	87.10%
El-Husseiny et al.	18	84 (60–120)	83.34%
Chen et al.	16	70 (38–103)	81.30%
Crawford et al.	41	66 (18–222)	80.50%
Shi et al.	14	67,4 (DS 27,9)	100%
Castagnini et al.	28	60 (24–144)	78.60%
Yishake et al.	28	48 (24–132)	85.7%
Current series	8	38.2 (24–132)	100%

## Data Availability

The data used to support the findings of the present study are available from the corresponding author upon request.
